# Emotion Recognition in Patients with Low-Grade Glioma before and after Surgery

**DOI:** 10.3390/brainsci12091259

**Published:** 2022-09-16

**Authors:** Anne M. Buunk, Marleen J. J. Gerritsen, Hanne-Rinck Jeltema, Michiel Wagemakers, Jan D. M. Metzemaekers, Rob J. M. Groen, Jacoba M. Spikman

**Affiliations:** 1 Department of Neurology, University Medical Center Groningen, University of Groningen, Hanzeplein 1, P.O. Box 30.001, 9700 RB Groningen, The Netherlands; 2 Department of Neurosurgery, University Medical Center Groningen, University of Groningen, Hanzeplein 1, P.O. Box 30.001, 9700 RB Groningen, The Netherlands

**Keywords:** low-grade glioma, social cognition, brain tumor, emotion recognition

## Abstract

Research on patients with low-grade gliomas (LGGs) showed neurocognitive impairments in various domains. However, social cognition has barely been investigated. Facial emotion recognition is a vital aspect of social cognition, but whether emotion recognition is affected in LGG patients is unclear. Therefore, we aimed to investigate the effect of LGG and resection by examining emotion recognition pre- and postoperatively. Additionally, the relationships among emotion recognition and general cognition and tumor location were investigated. Thirty patients with LGG who underwent resective surgery were included and matched with 63 healthy control participants (HCs). Emotion recognition was measured with the Facial Expressions of Emotion–Stimuli and Tests (FEEST) and general cognition with neuropsychological tests. Correlations and within-group and between-group comparisons were calculated. Before surgery, patients performed significantly worse than the HCs on FEEST-Total and FEEST-Anger. Paired comparisons showed no significant differences between FEEST scores before and post-surgery. No significant correlations with general cognition and tumor location were found. To conclude, the results of this study indicate that the tumor itself contributes significantly to social cognitive dysfunction and that surgery causes no additional deficit. Impairments were not related to general cognitive deficits or tumor location. Consequently, incorporating tests for emotion recognition into the neuropsychological assessment of patients with LGG is important.

## 1. Introduction

Low-grade gliomas (LGGs) are primary brain tumors, with a malignancy grade of I or II according to the World Health Organization’s (WHO) definition [1]. Standard care consists of neurosurgical intervention to reduce tumor mass and establish a histological diagnosis, often followed by radiotherapy and chemotherapy. The majority of patients with LGG are relatively young at the time of diagnosis, with a peak incidence in adults between 30 and 40 years of age [2]. Due to improved treatment methods, survival rates have increased [3,4], and consequently many patients will experience long-term cognitive, emotional, and behavioral problems interfering with daily functioning and societal participation. In patients with LGG, studies have demonstrated cognitive deficits in several neuropsychological domains: memory, attention, speed, executive function, and language [5,6].

However, until now, social cognition has barely been a topic of investigation in patients with LGG, despite the fact that this has recently been acknowledged as an important neurocognitive domain, which is known to be vital for daily life functioning [7]. Social cognition is the ability to process social information and react adequately in social situations, which is crucial for the maintenance of meaningful social relationships [8,9]. The recognition of emotional states of others, i.e., emotion recognition, is a central aspect of social cognition. In particular, facial expressions of emotions contain crucial information necessary to understand others’ state of mind. A few studies examined facial emotion recognition and other aspects of social cognition in mixed groups of patients with different types of brain tumors or selective groups of patients with a tumor in a specific brain region only (for example, insular tumors) [10,11,12]. To date, it is unclear to what extent facial emotion recognition is affected in patients with LGG, as detailed conclusions on LGG are not possible based on previous work. Moreover, the sole influence of the tumor on emotion recognition in patients with LGG is unknown, as previous literature about both pre- and postsurgery social cognition is also scarce [13,14]. Specifically, only one study [13] reported scores before the resection of patients with LGGs, high-grade gliomas (HGGs), and meningiomas separately. The results suggested intact emotion recognition in most patients presurgery and minor deficits in the acute phase postsurgery for LGG, which recovered largely to premorbid levels within a few months. Impairments in neurocognition, including social cognition, can be caused by the tumor, its treatment, or both. Thus, to determine if emotion recognition impairment is already present before treatment and hence a direct consequence of the tumor, the present study investigates emotion recognition both before and after surgery. 

Accordingly, if social cognitive impairments are already present before surgery, they might be influenced by different disease-related factors. For instance, the effects of tumor location and volume on general cognitive functioning have been described in patients with LGG [5]. Social cognition deficits have been related to damage in several brain circuits, including the orbitofrontal cortex, amygdala, and temporoparietal areas [15,16,17]. In addition, in patients with brain tumors, a distributed cortical–subcortical network has been suggested to facilitate emotion recognition [18]. Furthermore, it is plausible that the hemispheric location of the tumor influences social cognitive performance, but the results are inconclusive; some authors showed a worse performance in right-hemispheric patients [19], while others found left-hemispheric patients to be more impaired [14]. Thus, when investigating the effects of resection on social cognition, relevant disease-related variables need to be taken into account, as the precise associations in patients with LGG are not clear.

Furthermore, an important question is the extent to which performance on social cognition tests is influenced by general cognitive deficits and, consequently, if impaired performance on these tests indicates a deficit in social cognition exclusively. Measuring social cognition is difficult as tests are complex and tap several other cognitive functions, such as memory or attention. To measure emotion recognition, usually photographs of emotional expressions are presented that have to be recognized within a few seconds. It is likely that such a task also requires intact attention and mental speed in order to process the information quickly. Consequently, considering the wide range of possible cognitive deficits in patients with LGG [6], it is important to investigate to what extent they are related to performance on social cognition tests. To our knowledge, this association between general and social cognition has not been examined in patients with LGG until now. 

The aim of the present longitudinal study was to investigate the effect of glioma resection on emotion recognition in patients with LGG by examining the recognition of facial expressions both pre- and postoperatively. Subsequently, in the case of presurgery impairments, the aim was to investigate the relationship between social cognition and both general cognition and tumor location. 

## 2. Materials and Methods

### 2.1. Participants and Procedure

A cohort of Dutch patients who underwent resective surgery for LGG and neuropsychological assessment as part of routine clinical follow-up in the University Medical Center of Groningen (UMCG) between 2010 and 2018 was included in the study. Tumor grade was confirmed as being WHO grade II [20] by postoperative neuropathological analysis. Exclusion criteria were previous medical treatments (radiotherapy, chemotherapy, and surgery) and a history of neurological conditions or psychiatric disturbances. Tumor localization was determined by a neuroradiologist on magnetic resonance imaging (MRI) scans and categorized as mainly frontal, temporal, parietal, or subcortical. The histological tumor type (astrocytoma, oligodendroglioma, or oligoastrocytoma) was determined by a neuropathologist based on the tissue specimen obtained during the surgical procedure. 

All participants underwent a preoperative neuropsychological assessment between 1 and 3 months before surgery (T1). The same test battery was performed again at least 2 months postsurgery (T2). Healthy controls (HCs) were selected from a larger group of controls (collected in the context of studies at the UMCG, subdepartment Neuropsychology) and matched with the patients. The Medical Ethics Committee of the UMCG gave clearance to execution of the study. Written informed consent was not obliged, as data were obtained as part of routine clinical care.

### 2.2. Measures

#### 2.2.1. Social Cognition

Emotion recognition. The Ekman 60 Faces Test [21] is part of the Facial Expressions of Emotion–Stimuli and Test (FEEST) and was used to measure facial emotion recognition. During the task, participants had to decide which of six basic emotions (anger, disgust, anxiety, happiness, sadness, and surprise) best describes the facial expressions in 60 photographs. The scores per emotion range from 0–10 (FEEST-Anger, FEEST-Disgust, FEEST-Anxiety, FEEST-Happiness, FEEST-Sadness, and FEEST-Surprise), and the overall score ranged from 0–60 (FEEST-Total). 

#### 2.2.2. General Cognition

Memory. The Dutch version of the Rey Auditory Verbal Learning Test (15-Word Test, 15WT [22]) was used to measure immediate recall from memory. A series of 15 words weres presented to the participant, who had to reproduce as many words as possible. This was done in 5 trials, with a maximum score of 75. 

Attention, speed, and executive functions. The Trail Making Test (TMT [23]) versions A and B were used to measure mental speed and cognitive flexibility. Participants were asked to connect numbers (A) or alternating numbers and letters (B) in ascending order, as quickly as possible. Total scores are the number of seconds needed to complete parts A and B. 

Language. The category fluency (subtest Groninger Intelligence Test, GIT [24]) measures verbal fluency. Participants needed to name as many words as possible in one minute, belonging to a certain category (animals).

### 2.3. Statistical Analysis

All statistical analyses were performed using the Statistical Package for the Social Sciences version 23.0. To describe educational level, the Dutch classification system of Verhage was used [25], ranging from 1 (primary school) to 7 (university education). Descriptive statistics were used to describe demographic and disease characteristics. Chi-square and independent *t*-tests were used to compare demographic characteristics of the HCs and patients. To test for differences in FEEST scores between several (sub)groups (patients vs. HCs, frontal vs. a nonfrontal tumor, and right-hemispheric vs. left-hemispheric tumor), independent *t*-tests or (in the case of not normally distributed data) Mann–Whitney U tests were used. Cohen’s *d* was used to report effect sizes for all between-group comparisons (≤0.2 small effect, 0.2–0.5 medium effect, and >0.5 large effect). FEEST scores could also be examined in contrast to normative data, as norms were available, and performances below the tenth percentile were considered to be impaired [26]. Paired-sample *t*-tests and Wilcoxon signed-rank tests were performed to assess differences in FEEST scores over time. Standardized scores on tests for general cognition were compared to a normative sample (M = 50 and SD = 10) with one-sample *t*-tests. Pearson’s and Spearman’s correlations were used to assess associations between the FEEST and tests for general cognition. Bonferroni–Holm corrections were performed in the case of multiple comparisons. 

## 3. Results

Thirty patients with LGG were included, and 26 patients completed both T1 and T2; one patient could no longer participate due to tumor progression, and three patients cancelled their follow-up appointment. A group of 63 HCs did not differ from the patients with LGG with respect to age (*t* = −0.14, *p* > 0.05), sex (χ = 0.28, *p* > 0.05), and education (U = 898.5, *p* > 0.05), see Table 1.

### 3.1. Pre- and Postoperative Emotion Recognition

At T1, patients performed significantly worse on the FEEST-Total and FEEST-Anger compared to HCs, with a moderate to large effect size. No significant differences were found between patients with LGG and the HCs for the other emotions, see Table 2. 

The mean time between surgery and postoperative assessment was 6.3 months (SD = 4.2, median: 4.5). As shown in Table 3, paired comparisons showed no significant differences between scores on the FEEST before and scores on the FEEST after surgery. Before resection, 26.7% of all patients performed below the 10th percentile and thus showed an impairment in emotion recognition. After surgical resection, 19.2% was impaired. 

### 3.2. Relation between Emotion Recognition and General Cognition

To examine if deficits in general cognition were related to the performance on the FEEST, first the standardized scores on tests for general cognition were compared to the normative data (Table 4). One-sample *t*-tests showed only a significant difference on the 15WT; the patient group had a mean of 5.13 points below that of the normative group, meaning a mean of 0.51 standard deviations lower than the normative group. Subsequently, Pearson’s correlation between FEEST total and 15WT was calculated, which was small and nonsignificant (*r* = 0.26, *p* = 0.17).

### 3.3. Relation between Emotion Recognition and Tumor Location

Comparing patients with left- versus right-hemisphere LGG revealed no significant differences in FEEST scores (all *p*s > 0.05, Table 5). The effect sizes were large for the FEEST-Total, FEEST-Fear, and FEEST-Sadness scores, with lower scores in patients with a left-hemispheric tumor. In addition, no significant differences were found between patients with a frontal LGG and non-frontal LGG on the FEEST (all *p*s > 0.05), with small to medium effect sizes. 

## 4. Discussion

This study is the first to investigate the influence of tumor and tumor resection on social cognition in patients with LGG. We found impairments in emotion recognition, a crucial aspect of social cognition. These impairments were already present before surgery and did not significantly change afterward, indicating that these were caused by the tumor itself and that surgery had no additional detrimental effect in these cases. Furthermore, preoperative social cognitive deficits were not related to tumor location and could not be explained by deficits in general cognition.

To the best of our knowledge, this is the first study to examine emotion cognition in a sample of patients with LGG only, both before and after tumor resection. Prior to surgery, patients with LGG performed significantly worse on emotion recognition than a group of matched healthy controls, indicating that social cognitive impairments were induced by the tumor itself. Furthermore, using the available norm scores for the FEEST, the prevalence rate of emotion recognition deficits before resection was 26.7%. Therefore, our findings show a preoperative lower performance of the tumor group, with more than a quarter of these patients already performing on an impaired level. Based on previous studies, detailed conclusions about emotion recognition in patients with LGG had not been possible, as patients with different types of brain tumors (gliomas, meningiomas, and brain metastases) were described as one group. Considering the differences in possible consequences, mainly in severity and prognosis, it is important to investigate these different patient groups separately. Only one study analyzed the performance of patients with LGG as a subgroup, and, in contrast to our findings, found no indication for emotion recognition deficits in the preoperative phase [13]. A possible explanation for this may be the use of different neuropsychological tests. Campanella and colleagues used an experimental task to measure emotion recognition, containing 36 photographs of the six basic emotions. In our study, we used the FEEST that has been shown to be well-validated and reliable in various patient groups [27,28,29,30,31] and is therefore probably more sensitive to detect emotion recognition deficits in patients with LGG. Furthermore, our study was the first to investigate the distinct basic emotions in addition to overall emotion recognition in patients with LGG. Examining emotions separately in addition to overall emotion recognition is important, because impairments in specific basic emotions can be related to certain behavioral disturbances, as has been found in other neurological patient groups [27,32]. Our results show that the patients performed significantly worse compared to the healthy controls in the recognition of anger only. This can be of importance for daily functioning, considering the fact that impaired anger recognition has been associated with impaired self-awareness and behavioral problems as rated by spouses in stroke and TBI [27,33,34].

Not only were emotion recognition deficits already present before surgery, we also found no significant differences between pre- and postoperative emotion recognition. Therefore, the tumor appears to be the main cause of social cognitive impairments, not the surgery. This was unclear for social cognition in patients with LGG until now, but it is in line with previous results on general cognitive impairment before and after tumor resection [35,36]. Signaling the presence of emotion recognition deficits, especially before surgery, is important in educating patients with LGG and their spouses about possible consequences of their disease and preparing them for resection. Given the possible strong negative impact of impaired emotion recognition on daily functioning and societal participation, in particular on the fulfillment of social roles and the maintenance of meaningful social relationships, a timely identification is crucial. Additionally, our findings lead to the conclusion that no significant deterioration of social cognitive functions is to be expected in the subacute phase after surgery. This is not only an important factor when advising patients about the treatment plans and options, but also an indication that retesting social cognition in the first few months post-surgery is not imperative. Patients often experience emotional distress and fatigue in this phase; they have to deal with the fact that they have an incurable disease, while concurrently undergoing adjuvant treatment. Consequently, additional appointments in the hospital for diagnostic follow-up that are not useful, should be avoided in this phase. Notably, a neuropsychological assessment might be useful in later stages, when the decline of cognitive functions is possible, due to radiotherapy, chemotherapy, or tumor progression [5,35,36,37,38]. In addition, different types of resections (awake vs. asleep) were performed in our patient group, and no comparisons were made between the different types of resections due to the small numbers. Therefore, no conclusions about the possible effect of a supratotal resection on emotion recognition can be made based on the present study. More research on social cognition after supratotal resection, often performed in awake craniotomy, is needed, as is more knowledge on the intraoperative measurement of social cognition [39,40].

To our knowledge, this is the first study to investigate if the performance on emotion cognition tests before resection of the LGG was related to the general cognition and tumor location. First, our results show no impairments on the majority of tests for general cognition; i.e., attention, mental speed, and executive functions were intact on a group level. Only a deficit in verbal memory was found, and this deficit was not associated with poor performance on the test for emotion recognition. This lack of association and the fact that emotion recognition was impaired in more than a quarter of our patient group, whereas most general cognitive functions were intact, leads us to the conclusion that emotion recognition deficits are distinct from impairments in general cognitive functions (that is, memory impairment) in patients with LGG. Based on this conclusion and the number of patients with impaired emotion recognition in our group, we would recommend the application of a validated test for emotion recognition in routine neuropsychological assessments. Therewith, our results endorse the conclusions of Goebel et al. [12] who recommended the inclusion of social cognitive measures in the assessment of brain tumor patients. As noted before, including a social cognition measure at one time point, preferably before surgery, should be sufficient. 

Regarding the tumor location and social cognition, no differences in emotion recognition performance were found between patients with a frontal vs. a non-frontal tumor. LGGs are unique in the sense that they cause slow-growing lesions and are known to frequently involve the frontal lobe. However, because of this slow process of tumor growth, reorganization might occur, and the transference of functions takes place. This type of reorganization is often referred to as dynamical plasticity: the ability of brain networks to redistribute dynamical behavior over intact areas after a focal injury, such as a tumor [41]. This may partly explain the lack of differences in social cognitive performance between patients with LGG at different locations. Additionally, this possible reorganization in a process of slow growth may also explain the fact that no significant differences between patients with a left- and right-hemispheric tumor were found. This is in contrast with findings in patients with acute and sudden damage, such as a stroke, showing more severe impairments in emotion recognition in patients with right-hemispheric lesions [42]. In addition, the present results seem to be in line with the leading hypothesis that social cognition depends on the integrity of a broader frontotemporoparietal network, involving both hemispheres rather than solely on specific, isolated (frontal) brain areas [15,18]. Consequently, lesions in different areas of this network can lead to social cognition deficits, and emotion recognition should be assessed irrespective of tumor lateralization or location. 

Some limitations of our study should be mentioned. First, patients with gliomas in the left hemisphere were overrepresented in our group. Comparing social cognitive performance between left- and right-localized tumors did not show any significant differences; however, some effect sizes of these differences were large. A lack of power may have led to the present results; thus, further work including equally large groups of left- and right-hemispheric patients is needed. The same is true for comparisons between different types of resections (awake vs. asleep), which were not calculated due to the small numbers. Of note, these different types of resections also differ regarding aims and consequences. Awake surgery is aimed at reaching the best balance between the extent of the resection and the risks of deficits performed when appropriate, i.e., when gliomas extend into eloquent areas. This often leads to a supratotal resection, whereas this is not the case for surgery under general anesthesia. Secondly, our patient group was relatively small, limiting the investigation of the association between emotion recognition and specific lesion locations. A rather crude distinction between frontal and nonfrontal lesions was made and more precise mapping of lesions by using voxel-based lesion symptom mapping might clarify the role of specific regions in the social cognition in patients with LGG. Furthermore, whereas a conventional MRI can detect structural brain damage, more advanced brain imaging techniques might also be useful in investigating the neural underpinnings of social cognition deficits in LGG. For example, in future studies, techniques, such as diffusion tensor imaging, could be used to examine brain connectivity changes in LGG patients. Additionally, considering previously found associations between tumor volume and general cognition [5], further research on social cognition in patients with LGG may include tumor volume as a factor. Furthermore, the World Health Organization’s (WHO) 2007 classification was used in the present study, in which gliomas are divided into four grades based on their histopathology [43]. More recently, it has been suggested that specific genetic markers might better represent the tumor growth rate [20] and consequently cognitive impairments. For example, fewer cognitive deficits are found in patients with an Isocitrate Dehydrogenase (IDH) 1 or 2 mutation compared to patients with an IDH1 wild-type tumor [44]. Unfortunately, information on genetic mutations was not available for all patients in the present patient group. Lastly, because of the explorative nature of our study, the influence of epilepsy, use of antiepileptic drugs, mood disorders, and fatigue on emotion recognition was not investigated, although these factors are relevant in patients with brain tumors and can impact general cognition [45,46]. Future studies may shed light on the role of these factors regarding social cognition in patients with LGG.

## 5. Conclusions

In our group of patients with LGG, impairments in emotion recognition were found before surgery that did not worsen after resection. This supports the hypothesis that the tumor itself contributes significantly to social cognitive dysfunction. Furthermore, this impairment in emotion recognition could not be explained by deficits in general cognition and was not related to the tumor’s location. Consequently, incorporating tests for emotion recognition into the neuropsychological assessment of all patients with LGG is important, as it is crucial for appropriate psychoeducation. It is probable that emotion recognition deficits are related to behavioral problems in patients with LGG and consequently have a negative impact on daily functioning, but this topic is still in need of further investigation.

## Figures and Tables

**Table 1:** Characteristics of patient group on T1 and healthy controls.

	Patients N = 30	Healthy Controls N = 63
**Demographic characteristics**		
Sex, number of women (%)	17 (56.7%)	32 (50.8%)
Age on T1 (M ± SD)	41 ± 11	40.6 ± 13.9
Educational level (M ± SD)	5 ± 1	5 ± 1
**Treatment characteristics**		
Type of craniotomy, awake	16 (53.3%)	
*Postoperative course*		
Hemorrhage ^a^	0 (0%)	
Increase in neurological deficits ^b^	10 (33.3%)	
Wound infection	0 (0%)	
Epilepsy ^c^	5 (5.2%)	
**Disease characteristics**		
*Histopathology*		
Astrocytoma	16 (53.3%)	
Oligodendrocytoma	6 (20%)	
Oligoastrocytoma	8 (26.7%)	
*Location of LGG*		
Frontal	17 (56.7%)	
Temporal	10 (33.3%)	
Parietal	2 (6.7%)	
Subcortical	1 (3.3%)	
*Lateralization*		
Left	21 (70%)	
Right	9 (30%)	

Abbreviations: LGG, low-grade glioma; T1: preoperative neuropsychological assessment; ^a^ Hemorrhage for which recraniotomy was needed. ^b^ Increase compared to preoperative status, in first month post-surgery. In this group: (mostly temporary) speech or language difficulties, quadrantanopsia, and motor deficits. ^c^ Two or more seizures after surgery or significant increase in seizures compared to preoperative status.

**Table 2:** Comparison between LGG patients and healthy controls on tests for emotion recognition at T1.

	HCs	LGG			
	N = 63	N = 30	T/Z	*p*	Cohen’s *d*
**Emotion recognition**					
FEEST total	49.5 (4.9)	46.9 (5.4)	2.36	**0.02 *^a^***	0.52
Anger	8.3 (1.5)	7.6 (1.9)	−2.06	**0.04 *^a^***	0.43
Disgust	7.8 (1.9)	7.3 (1.9)	−1.20	0.23	0.27
Fear	6.7 (2.2)	6.0 (2.2)	−1.60	0.11	0.32
Happiness	10.0 (0.2)	9.9 (0.3)	−0.96	0.33	0.43
Sadness	7.8 (1.8)	7.2 (2.2)	−0.91	0.37	0.31
Surprise	8.9 (1.3)	8.9 (1.4)	−0.73	0.47	0

Abbreviations: LGG, low-grade glioma; HCs, healthy controls; FEEST, Facial Expressions of Emotion—Stimuli and Tests. Note: An independent *t*-test was used to compare FEEST total scores; Mann–Whitney U tests were used to compare scores on FEEST subtests. T1: preoperative neuropsychological assessment. *^a^* Means differ significantly between groups, *p* < 0.05.

**Table 3:** Paired comparisons between preoperative and postoperative scores on test for emotion recognition.

	T1	T2	Z/t	*p*
	M (SD) N = 26	M (SD) N = 26		
FEEST total	47.4 (4.4)	47.4 (5.6)	0.00	1.00
Anger	7.6 (1.9)	8.2 (1.5)	−1.59	0.11
Disgust	7.3 (1.9)	7.3 (2.0)	−0.10	0.92
Fear	6.1 (2.2)	5.6 (2.5)	0.64	0.52
Happiness	9.9 (0.3)	10.0 (0.2)	−1.00	0.32
Sadness	7.6 (1.5)	7.5 (2.1)	−0.25	0.81
Surprise	8.9 (1.2)	8.8 (1.0)	−0.40	0.69

Abbreviations: LGG, low-grade glioma; HCs, healthy controls; FEEST, Facial Expressions of Emotion—Stimuli and Tests. Note: Paired sample *t*-tests were used to compare FEEST total scores; Wilcoxon’s signed-rank tests were used to compare scores on FEEST subtests. T1: preoperative neuropsychological assessment, T2: postoperative neuropsychological assessment.

**Table 4:** Scores on tests for general cognition at T1 (N = 30) and comparison with normative means.

	Raw Score M (SD)	Standardized Score M (SD)	Mean Difference with Normative Group	*T*	*p*
**Memory**					
15WT	46.4 (9.2)	44.9 (10.1)	−5.13	−2.79	**0.009 *^a^***
**Attention and executive functions**					
TMT-A	31.8 (9.7)	47.3 (8.9)	−2.67	−1.64	0.11
TMT-B	69.2 (30.4)	49.2 (9.7)	−0.77	−0.43	0.67
**Language**					
Fluency	23.1 (4.7)	47.8 (8.3)	−2.20	−1.46	0.16

Abbreviations: 15WT, 15-Word Test; TMT-A, Trail Making Test version A; TMT-B, Trail Making Test version B. Note: One-sample *t*-tests were used to compare the standardized scores with the normative group (M = 50, SD = 10). T1: preoperative neuropsychological assessment. *^a^* Means differ significantly between groups, *p* < 0.05.

**Table 5:** Emotion recognition scores for patients with left- and right-sided LGG and frontal and nonfrontal LGG at T2.

	Left M (SD) (N = 21)	Right M (SD) (N = 9)	*Z*	Cohen’s *d*	Frontal M (SD) (N = 13)	Non-Frontal M (SD) (N = 17)	*Z*	Cohen’s *d*
FEEST	46.1 (5.3)	48.7 (5.3)	−1.23	0.51	47.4 (5.6)	46.1 (5.1)	−1.07	0.25
Anger	7.7 (1.6)	7.3 (2.4)	−0.09	0.22	7.8 (1.6)	7.2 (2.2)	−0.54	0.32
Disgust	7.1 (1.8)	7.9 (2.1)	−1.15	0.44	7.4 (2.0)	7.2 (1.9)	−0.19	0.11
Fear	5.5 (2.3)	7.0 (1.7)	−1.69	0.72	5.9 (2.4)	6.0 (2.0)	−0.11	0.05
Happiness	9.9 (0.3)	9.9 (0.3)	−0.31	0.00	9.9 (0.3)	9.9 (0.3)	−0.36	0.00
Sadness	6.9 (2.5)	8.0 (1.0)	−1.05	0.52	7.4 (2.0)	7.1 (2.6)	−0.26	0.13
Surprise	9.0 (0.9)	8.6 (1.6)	−0.40	0.26	9.0 (1.1)	8.7 (1.2)	−0.90	0.27

Abbreviations: LGG, low-grade glioma; FEEST, Facial Expressions of Emotion—Stimuli and Tests. Note: Mann–Whitney U tests were used to compare FEEST scores between groups. T2: postoperative neuropsychological assessment.

## Data Availability

The data that support the findings of this study are available from the corresponding author, A.M.B., upon reasonable request.

## References

[1] Louis D.N., Perry A., Wesseling P., Brat D.J., Cree I.A., Figarella-Branger D., Hawkins C., Ng H.K., Pfister S.M., Reifenberger G. (2021). The 2021 WHO Classification of Tumors of the Central Nervous System: A Summary. Neuro. Oncol..

[2] Ostrom Q.T., Bauchet L., Davis F.G., Deltour I., Fisher J.L., Langer C.E., Pekmezci M., Schwartzbaum J.A., Turner M.C., Walsh K.M. (2014). The Epidemiology of Glioma in Adults: A “State of the Science” Review. Neuro. Oncol..

[3] Roelz R., Strohmaier D., Jabbarli R., Kraeutle R., Egger K., Coenen V.A., Weyerbrock A., Reinacher P.C. (2016). Residual Tumor Volume as Best Outcome Predictor in Low Grade Glioma—A Nine-Years Near-Randomized Survey of Surgery vs. Biopsy. Sci. Rep..

[4] Capelle L., Fontaine D., Mandonnet E., Taillandier L., Golmard J.L., Bauchet L., Pallud J., Peruzzi P., Baron M.H., Kujas M. (2013). Spontaneous and Therapeutic Prognostic Factors in Adult Hemispheric World Health Organization Grade II Gliomas: A Series of 1097 Cases: Clinical Article. J. Neurosurg..

[5] Van Kessel E., Emons M.A.C., Wajer I.H., van Baarsen K.M., Broekman M.L., Robe P.A., Snijders T.J., Van Zandvoort M.J.E. (2019). Tumor-related neurocognitive dysfunction in patients with diffuse glioma: A retrospective cohort study prior to antitumor treatment. Neurooncol. Pract..

[6] Van Loon E.M.P., Heijenbrok-Kal M.H., van Loon W.S., van den Bent M.J., Vincent A.J.P.E., de Koning I., Ribbers G.M. (2015). Assessment Methods and Prevalence of Cognitive Dysfunction in Patients with Low-Grade Glioma: A Systematic Review. J. Rehabil. Med..

[7] Henry J.D., von Hippel W., Molenberghs P., Lee T., Sachdev P.S. (2016). Clinical Assessment of Social Cognitive Function in Neurological Disorders. Nat. Rev..

[8] Adolphs R. (2009). The Social Brain: Neural Basis of Social Knowledge. Annu. Rev. Psychol..

[9] Amodio D.M., Frith C.D. (2006). Meeting of Minds: The Medial Frontal Cortex and Social Cognition. Nat. Rev..

[10] Campanella F., Shallice T., Ius T., Fabbro F., Skrap M. (2014). Impact of Brain Tumour Location on Emotion and Personality: A Voxel-Based Lesion-Symptom Mapping Study on Mentalization Processes. Brain.

[11] Chen P., Wang G., Ma R., Jing F., Zhang Y., Wang Y., Zhang P., Niu C., Zhang X. (2016). Multidimensional Assessment of Empathic Abilities in Patients with Insular Glioma. Cogn. Affect. Behav. Neurosci..

[12] Goebel S., Mehdorn H.M., Wiesner C.D. (2018). Social Cognition in Patients with Intracranial Tumors: Do We Forget Something in the Routine Neuropsychological Examination?. J. Neurooncol..

[13] Campanella F., Fabbro F., Ius T., Shallice T., Skrap M. (2015). Acute Effects of Surgery on Emotion and Personality of Brain Tumor Patients: Surgery Impact, Histological Aspects, and Recovery. Neuro. Oncol..

[14] Mattavelli G., Pisoni A., Casarotti A., Comi A., Sera G., Riva M., Bizzi A., Rossi M., Bello L., Papagno C. (2019). Consequences of Brain Tumour Resection on Emotion Recognition. J. Neuropsychol..

[15] Adolphs R. (2001). The Neurobiology of Social Cognition. Curr. Opin. Neurobiol..

[16] Heberlein A.S., Padon A.A., Gillihan S.J., Farah M.J., Fellows L.K. (2008). Ventromedial Frontal Lobe Plays a Critical Role in Facial Emotion Recognition. J. Cogn. Neurosci..

[17] Shamay-Tsoory S.G. (2011). The Neural Bases for Empathy. Neuroscientist.

[18] Pertz M., Okoniewski A., Schlegel U., Thoma P. (2020). Impairment of Sociocognitive Functions in Patients with Brain Tumours. Neurosci. Biobehav. Rev..

[19] Luherne-du Boullay V., Plaza M., Perrault A., Capelle L., Chaby L. (2014). Atypical Crossmodal Emotional Integration in Patients with Gliomas. Brain Cogn..

[20] Louis D.N., Perry A., Reifenberger G., von Deimling A., Figarella-Branger D., Cavenee W.K., Ohgaki H., Wiestler O.D., Kleihues P., Ellison D.W. (2016). The 2016 World Health Organization Classification of Tumors of the Central Nervous System: A Summary. Acta Neuropathol..

[21] Young A., Perrett D., Calder A., Sprengelmeyer R., Ekman P. (2002). Facial Expressions of Emotion—Stimuli and Tests (FEEST).

[22] Deelman B.G., Brouwer W.H., van Zomeren A.H., Saan R.J., Jennekens-Schinkel A., Diamant J.J., Diesfeldt H.F.A., Haaxma R. (1980). Functiestoornissen Na Trauma Capitis. Neuropsychologie in Nederland.

[23] Reitan R.M. (1958). Validity of the Trail Making Test as an Indicator of Organic Brain Damage. Percept. Mot. Ski..

[24] Luteijn F., Barelds D.P.F. (2004). GIT-2 Groninger Intelligentietest.

[25] Verhage F. (1964). Intelligentie En Leeftijd: Onderzoek Bij Nederlanders van Twaalf Tot Zevenenzeventig Jaar [Intelligence and Age: Study on Dutch People from Age 12 to 77].

[26] Lezak M.D., Howieson D.B., Loring D.W., Hannay H.J., Fischer J.S. (2004). Neuropsychological Assessment.

[27] Nijsse B., Spikman J.M., Visser-Meily J.M.A., de Kort P.L.M., van Heugten C.M. (2019). Social Cognition Impairments Are Associated with Behavioural Changes in the Long Term after Stroke. PLoS ONE.

[28] Voncken L., Timmerman M.E., Spikman J.M., Huitema R.B. (2018). Beschrijving van de Nieuwe, Nederlandse Normering van de Ekman 60 Faces Test (EFT), Onderdeel van de FEEST [Description of the New, Dutch Norms of the Ekman 60 Faces Test (EFT), Part of the FEEST]. Tijdschr. Voor Neuropsychol..

[29] Spikman J.M., Timmerman M.E., Milders M.V., Veenstra W.S., van der Naalt J. (2012). Social Cognition Impairments in Relation to General Cognitive Deficits, Injury Severity, and Prefrontal Lesions in Traumatic Brain Injury Patients. J. Neurotrauma.

[30] Aben H.P., Visser-Meily J.M., Biessels G.J., de Kort P.L., Spikman J.M. (2020). High Occurrence of Impaired Emotion Recognition after Ischemic Stroke. Eur. Stroke J..

[31] Buunk A.M., Spikman J.M., Veenstra W.S., van Laar P.J., Metzemaekers J.D.M., van Dijk J.M.C., Meiners L.C., Groen R.J.M. (2017). Social Cognition Impairments after Aneurysmal Subarachnoid Haemorrhage: Associations with Deficits in Interpersonal Behaviour, Apathy, and Impaired Self-Awareness. Neuropsychologia.

[32] Visser-Keizer A.C., Westerhof-Evers H.J., Gerritsen M.J.J., Der Van Naalt J., Spikman J.M. (2016). To Fear Is to Gain? The Role of Fear Recognition in Risky Decision Making in TBI Patients and Healthy Controls. PLoS ONE.

[33] Spikman J.M., Milders M.V., Visser-Keizer A.C., Westerhof-Evers H.J., Herben-Dekker M., van der Naalt J. (2013). Deficits in Facial Emotion Recognition Indicate Behavioral Changes and Impaired Self-Awareness after Moderate to Severe Traumatic Brain Injury. PLoS ONE.

[34] Neumann D., Malec J.F., Hammond F.M. (2015). The Association of Negative Attributions with Irritation and Anger after Brain Injury. Rehabil. Psychol..

[35] Klein M., Heimans J.J., Aaronson N.K., van der Ploeg H.M., Grit J., Muller M., Postma T.J., Mooij J.J., Boerman R.H., Beute G.N. (2002). Effect of Radiotherapy and Other Treatment-Related Factors on Mid-Term to Long-Term Cognitive Sequelae in Low-Grade Gliomas: A Comparative Study. Lancet.

[36] Taphoorn M.J., Schiphorst A.K., Snoek F.J., Lindeboom J., Wolbers J.G., Karim A.B., Huijgens P.C., Heimans J.J. (1994). Cognitive Functions and Quality of Life in Patients with Low-Grade Gliomas: The Impact of Radiotherapy. Ann. Neurol..

[37] Douw L., Klein M., Fagel S.S., van den Heuvel J., Taphoorn M.J., Aaronson N.K., Postma T.J., Vandertop W.P., Mooij J.J., Boerman R.H. (2009). Cognitive and Radiological Effects of Radiotherapy in Patients with Low-Grade Glioma: Long-Term Follow-Up. Lancet. Neurol..

[38] Lawrie T.A., Gillespie D., Dowswell T., Evans J., Erridge S., Vale L., Kernohan A., Grant R. (2019). Long-Term Neurocognitive and Other Side Effects of Radiotherapy, with or without Chemotherapy, for Glioma. Cochrane Database Syst. Rev..

[39] Mandonnet E., Herbet G., Duffau H. (2020). Letter: Introducing New Tasks for Intraoperative Mapping in Awake Glioma Surgery: Clearing the Line Between Patient Care and Scientific Research. Neurosurgery.

[40] Prat-Acín R., Galeano-Senabre I., López-Ruiz P., Ayuso-Sacido A., Espert-Tortajada R. (2021). Intraoperative Brain Mapping of Language, Cognitive Functions, and Social Cognition in Awake Surgery of Low-Grade Gliomas Located in the Right Non-Dominant Hemisphere. Clin. Neurol. Neurosurg..

[41] Duffau H. (2012). The “Frontal Syndrome” Revisited: Lessons from Electrostimulation Mapping Studies. Cortex.

[42] Yuvaraj R., Murugappan M., Norlinah M.I., Sundaraj K., Khairiyah M. (2013). Review of Emotion Recognition in Stroke Patients. Dement. Geriatr. Cogn. Disord..

[43] Louis D.N., Ohgaki H., Wiestler O.D., Cavenee W.K., Burger P.C., Jouvet A., Scheithauer B.W., Kleihues P. (2007). The 2007 WHO Classification of Tumours of the Central Nervous System. Acta Neuropathol..

[44] Wefel J.S., Noll K.R., Rao G., Cahill D.P. (2016). Neurocognitive Function Varies by IDH1 Genetic Mutation Status in Patients with Malignant Glioma Prior to Surgical Resection. Neuro. Oncol..

[45] Klein M., Engelberts N.H.J., van der Ploeg H.M., Kasteleijn-Nolst Trenité D.G.A., Aaronson N.K., Taphoorn M.J.B., Baaijen H., Vandertop W.P., Muller M., Postma T.J. (2003). Epilepsy in Low-Grade Gliomas: The Impact on Cognitive Function and Quality of Life. Ann. Neurol..

[46] Van Coevorden-van Loon E.M.P., Coomans M.B., Heijenbrok-Kal M.H., Ribbers G.M., van den Bent M.J. (2017). Fatigue in Patients with Low Grade Glioma: Systematic Evaluation of Assessment and Prevalence. J. Neurooncol..

